# Acceptance of Illness and Health Behaviours of Patients with End-Stage Renal Disease Treated with Haemodialysis: A Single-Centre Study

**DOI:** 10.3390/healthcare12242562

**Published:** 2024-12-19

**Authors:** Marta Katarzyna Hreńczuk, Weronika Rudnicka, Tomasz Dawid Piątek, Piotr Małkowski

**Affiliations:** Department of Surgical and Transplantation Nursing and Extracorporeal Therapies, Faculty Health Sciences, Medical University of Warsaw, 02-007 Warsaw, Polandtomasz.piatek@wum.edu.pl (T.D.P.);

**Keywords:** end-stage renal disease, haemodialysis, illness acceptance, health behaviours

## Abstract

Background: Patients with end-stage renal disease undergoing haemodialysis experience numerous life changes often requiring significant sacrifices. Adaptation to the limitations associated with the disease and dialysis treatments may influence their health behaviour. Aim: The aim of the study was to analyse the relationship between the level of illness acceptance and the intensity of health behaviours in patients treated with haemodialysis. Methods: The study used a diagnostic survey method using the Acceptance of Illness Scale and Health Behaviour Inventory questionnaires, adapted to Polish conditions by Z. Juczyński. The study was conducted from December 2022 to March 2023 among 105 patients in one dialysis centre in Poland. Results: The patients showed an average level of disease acceptance (22.64 ± 6.52) and presented a low level of health behaviour intensity (79.11 ± 12.46). The disease acceptance index and the intensity of health behaviours showed positive correlations with age, disease duration, and the duration of haemodialysis treatment. Statistically significant positive correlations were identified between illness acceptance and the intensity of health-related behaviours. Conclusions: The acceptance of illness may influence adherence to health behaviours, which highlights the importance of introducing integrated and early therapeutic support to assist in the acceptance process.

## 1. Introduction

The acceptance of the disease and health behaviours of patients are key elements of effective treatment, management, and improvement of quality of life. End-stage renal disease (ESRD), which requires regular haemodialysis, poses not only physical challenges for the patients but also emotional and psychosocial ones. Knowing and understanding patients’ acceptance of the disease is important in planning care, as it may influence patient compliance and involvement in their therapy [1,2,3]. Many studies confirm a positive correlation between disease acceptance and quality of life in patients with chronic diseases [4,5,6,7]. Research shows that patients who demonstrate a greater level of acceptance of the disease tend to make better health decisions and better adapt to the difficult conditions of living with a chronic disease [1]. Reduced acceptance may lead to negative behaviours, which in turn increase the risk of complications and reduce the quality of life [8,9,10,11,12,13]. Moreover, psychological support and health education are essential to improve disease acceptance and the health behaviour of patients. Interventions that focus on developing coping skills and improving quality of life can bring measurable benefits [13,14,15]. As the number of patients with ESRD increases, understanding these dynamics becomes crucial to developing effective support strategies and therapies.

Health behaviours are conscious actions undertaken by individuals to maintain, improve, or protect their health. These behaviours significantly influence the health status of both individuals and populations. Their effectiveness depends on regularity, persistence, the level of personal engagement, and the diversity of actions taken. The level of health behaviours reflects the extent to which an individual commits to and consistently implements these actions [16,17].

Studies indicate that there is low health awareness among patients with chronic kidney disease (CKD), which significantly limits the extent to which patients are able to self-manage their disease and directly or indirectly influence their condition [4,14,18]. The aim of the study was to analyse the relationship between the level of illness acceptance and the intensification of health behaviours in patients with end-stage renal disease undergoing chronic haemodialysis treatment. Additionally, the study aimed to understand the impact of clinical and sociodemographic variables on these phenomena, and knowing these factors may contribute to the development and implementation of support interventions and consequently an improvement in the quality of life of this group of patients.

## 2. Materials and Methods

The study group included patients with end-stage renal disease who were treated chronically with haemodialysis. This study was conducted using a diagnostic survey method. The research tool used was a survey questionnaire consisting of two standardised questionnaires, namely the Acceptance of Illness Scale (AIS) and the Health Behaviour Inventory (HBI), which are part of the collection “Measurement Tools in Health Promotion and Psychology” by Zygfryd Juczyński. Additionally, the questionnaire was supplemented with a metric containing 12 questions aimed at characterising the study group in terms of selected sociodemographic factors (gender, age, education, place of residence, marital status, and social status) and clinical factors (comorbidities, adverse events occurring during haemodialysis and their frequency, duration of the disease, duration of haemodialysis treatment, and type of vascular access for haemodialysis).

The AIS is intended for testing adults and currently ill people. It was developed by Felton, Revenson, and Hinrichsen, and adapted to Polish conditions by Juczyński [19]. It is used to measure the degree of acceptance of the disease and adaptation to the limitations it imposes. It contains eight statements that describe the emotional consequences of poor health. They mainly concern the recognition of difficulties and limitations imposed by the disease, lack of independence, a sense of dependence on people around them, and a decrease in self-esteem. In each statement, the respondent describes their current state on a 5-point scale, from 1—“I strongly agree” to 5—“I strongly disagree”. A score of 1 indicates poor adaptation to the disease, while a score of 5 indicates full acceptance. The general scoring range is from 8 to 40 points, where a low score indicates a minimal level or even lack of acceptance and adaptation to the disease and the patient’s experience of negative emotions, while a high score indicates acceptance of the disease and no accompanying mental discomfort. There is a certain interpretation of the AIS in the literature, but it is not confirmed by the authors of the scale; nevertheless, it gives an idea of the level of acceptance of the disease. A score of 8–18 points indicates a low level (no acceptance), and a score of 19–29 points indicates a medium level, while a score above 29 is classified as a high level of acceptance [20]. This interpretation was used in the results.

The HBI contains 24 statements that describe positive health-related behaviours. The study allows for determining the overall intensity of health-promoting activities, as well as separately, in relation to four distinguished subscales of health-related behaviours, containing six items each. The person who completes the questionnaire indicates how often they undertook the activities listed in the last year, using a five-point scale: 1—almost never, 2—rarely, 3—from time to time, 4—often, and 5—almost always. By adding up the numerical values, it is possible to obtain an overall indicator of the intensity of health-related behaviours. The overall range of points is from 24 to 120, while in each category, the sum of points is divided by 6, giving a range from 6 to 30 points. In both cases, the higher the score, the proportionally greater the intensity of the declared HB. In order to interpret the overall severity index, it is converted into standardised units on the sten scale, according to which sten 1–4 represents low HB severity, 5–6 are average values, and sten 7–10 are high values [19,21].

The *t*-test for independent samples, one-way analysis of variance, and Pearson’s correlation coefficient (r) were used for statistical analysis. The values of the analysed measurable parameters were presented using the mean value (M), minimum, maximum, and standard deviation (SD), and for the non-measurable parameters using the number (N) and percentage (%). A statistical significance level (*p*) of <0.05 was assumed. All calculations were performed using the SPSS STATISTICS 22 package.

This study was conducted in accordance with the principles of the Helsinki Declaration, and the Bioethics Committee of the Medical University of Warsaw confirmed its compliance with the principles of ethical research (KBE/279/2022). The manager of the facility agreed to implement the study. The study inclusion criteria included a respondent age of ≥18 years, diagnosed with stage V chronic kidney disease, and undergoing haemodialysis treatment. Participation in the study was anonymous and voluntary; completing the survey questionnaire by the patient was equivalent to giving consent to participate. All patients treated at the Dialysis Centre met the inclusion criteria and were included in the study. Patients who were under the constant care of the centre constituted 81% of the respondents, while patients staying at the facility as part of guest dialysis constituted the remaining 19%.

The researchers were from outside the facility where the patients were treated. The completed questionnaires were placed in a special box, to which only the researcher had access.

## 3. Results

### 3.1. Study Group

The study included 105 ESRD patients undergoing haemodialysis at one of the DaVita Dialysis Centres in Poland. The duration of the study was from December 2022 to March 2023. The study group included 45 women (42.9%) and 60 men (57.1%), and the average age of the participants was 58.90 years. Higher education (37.1%) and secondary education (33.3%) predominated among respondents. The majority of participants (96.2%) were urban residents. Overall, 33.3% of respondents declared being in a relationship, and 46.7% were employed under a contract of employment.

In the clinical analysis, concomitant diseases were present in all patients, with the most frequently reported cases of hypertension and diabetes. Adverse events during haemodialysis (HD) occurred in 77.7% of the study participants, most frequently including muscle cramps and hypotension. The most commonly used vascular access for HD was an arteriovenous fistula. The mean duration of the ESRD counted since the diagnosis was 5.79 years, and the mean duration of haemodialysis treatment was 3.98 years (Table 1).

### 3.2. Acceptance of Illness

**Figure 1:**
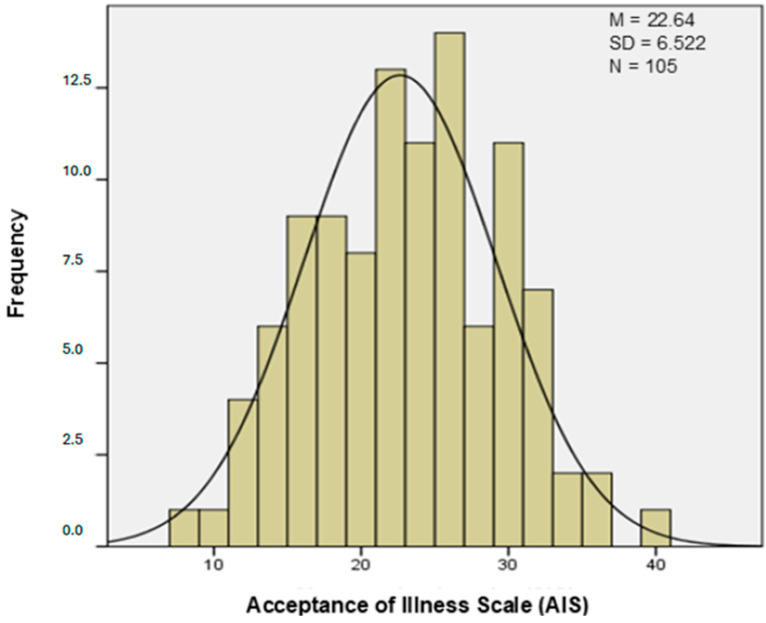
The range of results obtained on the AIS.

The average score of disease acceptance on the AIS among the respondents was 22.64 ± 6.52 points (Figure 1). In the study group, 17.1% of patients had a high level of acceptance, and 54.3% showed an average level, while 28.6% of the respondents did not accept their disease.

The average values for individual statements included in the AIS ranged from 2.23 points to 3.70 points. The highest level of disease acceptance was manifested in the feeling of being a valuable person and the lack of belief in being useless due to the disease. Problems with acceptance most often manifested themselves in difficulties in adapting to the limitations imposed by the disease (Table 2).

The level of acceptance of the disease was slightly higher among women (23.42 points) than among men (22.05 points), but this difference was not statistically significant (*p* > 0.05). The acceptance rate showed a significant correlation with the patients’ level of education. A higher level of disease acceptance was found in people with primary education. As the level of education increased, the degree of disease acceptance decreased. Rural residents reached a level of acceptance comparable to that of city dwellers. It was also found that people in a relationship had a significantly higher level of disease acceptance. The acceptance rate was also significantly related to professional status; a higher level of acceptance was found among retirees and pensioners, while significantly lower results were observed among professionally active and unemployed people (Table 3).

There was no significant relationship between the level of disease acceptance, and the presence and frequency of side effects during haemodialysis (*p* > 0.05). Comorbidities reported by the study participants also had no significant impact on the AIS scores. The type of vascular access was not a factor determining the degree of acceptance among haemodialysis patients (*p* > 0.05). Moreover, it was found that the acceptance rate correlated positively with the age of the respondents, the duration of the disease, and the duration of the haemodialysis treatment. Older people, patients who had been ill for a longer period, and patients who had been on renal replacement therapy for a longer period of time, showed a significantly higher level of acceptance (Table 4).

### 3.3. Health Behaviours

The overall health behaviour intensity index in the study group was on average 79.11 ± 12.46, which corresponded to a sten value of 5.16 ± 1.73. With respect to individual HBI categories, the highest scores were obtained in the positive mental attitude category, and the lowest scores were attributed to health practices. The results regarding preventive behaviours and proper eating habits were comparable (Table 5).

The analysis of the sten scale indicates that patients with end-stage renal disease undergoing haemodialysis presented a low (40.0%) or average (37.1%) level of intensity of health behaviours, while 22.9% of respondents obtained high results.

There were no significant differences in the intensity of health-related behaviours depending on gender or education level (*p* > 0.05). The results were higher among urban dwellers, especially in the categories of healthy eating habits, preventive behaviours, and positive mental attitude. Marital status was also found to influence health-related behaviours; people in a relationship achieved better results on all subscales. In the context of professional status, a higher intensity of health-related behaviours was observed among pensioners and retirees, and significant differences were observed in all HBI categories (Table 6).

Comorbidities significantly differentiated the patients’ health-related behaviours. People with diabetes showed a higher intensity of health behaviours, which translated into better results in terms of proper eating habits, preventive behaviours, and health practices. In turn, patients with cardiac arrhythmias had lower scores in the area of eating habits. People with other health conditions had higher health behaviour intensity scores, which were associated with better health practices (Table 7).

Factors such as the occurrence and frequency of adverse events during haemodialysis and the type of vascular access had no significant impact on health behaviour indicators (*p* > 0.05). As in the case of disease acceptance, the intensity of health behaviours correlated positively with age, disease duration, and haemodialysis treatment duration. A higher level of intensity of health-related behaviours in general and in individual dimensions concerned older people, those who had been ill for a longer period of time, and those treated with haemodialysis for a longer period of time (Table 8).

### 3.4. Level of Disease Acceptance and Intensity of Health Behaviours

This study found that the correlations between illness acceptance and the intensity of health behaviours were positive and statistically significant. A higher score on the AIS was associated with a proportionally higher index of health-related behaviours in general (r = 0.682) and in each of the HBI categories. Acceptance of the disease correlated the most with proper eating habits (r = 0.624) and the least with preventive behaviours (r = 0.550) (Table 9).

## 4. Discussion

Acceptance of illness is a psychological process that plays a key role in coping with chronic diseases; it means that the patient accepts the reality of their health situation, which may lead to better adaptation and a reduction in the stress associated with the disease [1,2,4,8]. According to this definition, acceptance does not mean resignation but enables patients to better adapt to reality. Acceptance can be a factor that supports a proactive approach to one’s health. In the context of ESRD, disease acceptance can significantly impact patients’ quality of life, allowing them to better cope with the daily challenges of treatment, such as regular dialysis [4]. Understanding the mechanisms of disease acceptance and its impact on health behaviours becomes important in the context of therapeutic interventions aimed at supporting patients with end-stage renal disease.

Data from this study indicate that the overall disease acceptance index in the group of ESRD patients reached an average level (22.64 points), suggesting moderate acceptance. Similar results were obtained by Pompey et al., who found that more than half of the participants had moderate acceptance (50.9%), and only 19.3% of patients showed full acceptance of the illness [4]. In this study, full acceptance was observed in 17.1% of patients. Oskędra et al. studied patients with multiple sclerosis, chronic obstructive pulmonary disease, and chronic heart failure, obtaining mean scores of 25.60 points, 28.56 points, and 27.76 points, respectively, which indicates moderate acceptance [22]. These results are higher than in this study. Low disease acceptance rates have also been observed in patients with cancer, which is a result of high levels of stress and anxiety [7,23,24,25]; those with epilepsy [26]; and patients diagnosed with mental disorders such as anxiety disorders, depression, and personality disorders [27]. Their results are comparable to those obtained for patients with ESRD, who often have difficulties in accepting the complex treatment and limitations related to the therapy resulting from chronic dialysis treatment [4]. High rates of disease acceptance are achieved by patients with diabetes, both type 1 and type 2. These patients, as well as patients with thyroid diseases, accept their disease well thanks to access to education and support, which promotes better control [6,10,13,28,29,30,31,32,33]. The literature review suggests that the issue of accepting a disease such as diabetes has been the most researched topic.

In this study, the overall health behaviour intensity index was on average 79.11 points. Better results were obtained in the study by Gniadek et al., in which the overall HB index was 90.56 points. The authors also stated that a positive mental attitude (23.29 points) outweighed the other categories [34]. The results of various studies, including this one, indicate a positive mental attitude of haemodialysis patients, who indicated avoiding depressing emotions and receiving support from their loved ones [34,35]. Similar results were obtained by Sień et al., who studied patients with thyroid diseases [32]. Marzec et al. studied patients with CKD, achieving an average score of 86.75 points. In their study, the highest results were obtained in the category of preventive behaviours (3.70 points), while health practices (3.46 points) were the least frequently declared [36]. In this study, a low level of health practices was also observed (3.18 points).

Understanding the impact of clinical and sociodemographic variables on disease acceptance and health behaviours is crucial and may be the basis for developing individualised support strategies that take into account the needs of patients with different levels of acceptance and health behaviours. Research indicates that many factors can significantly influence patients’ ability to accept their health situation and adhere to health-related behaviours. The results of this study suggest that the degree of disease acceptance and the health behaviour index correlated positively with the age of patients, the duration of the disease, and the duration of haemodialysis treatment. Older patients are more likely to demonstrate greater acceptance and better adherence to health behaviours, which may be a result of a longer experience with the chronic disease. Opposite results are presented by Łatka et al., who suggest that a longer duration of the haemodialysis treatment is associated with a lower level of acceptance, and age does not significantly affect the AIS [35]. A potential reason for this discrepancy could be the average duration of dialysis in the patients, which was 6.66 years in the study by Łatka et al., whereas it was half as long in our study. Patients undergoing long-term treatment may experience increasing burnout, frustration, and fatigue with the treatment process [37]. Over time, as the daily treatment routine imposes significant restrictions on the patient, acceptance of their health condition may decline. Patients may start to perceive haemodialysis as a permanent, inevitable part of life, which can lead to resistance and a decrease in positive acceptance of their situation. Other studies also indicate that the longer duration of the disease and its associated limitations may contribute to low levels of acceptance [12,13,38]. In the study by Gniadek et al., the duration of haemodialysis treatment did not have a significant impact on the level of health behaviours, which is not consistent with the results of this research [34]. In this analysis, it was noted that people who were in a relationship and those who were retired or receiving a disability pension achieved better results on both the AIS and HBI scores. This suggests that the support of loved ones and the organisation of free time are of key importance in the process of acceptance of the disease and motivation to take health-related activities. It is interesting that, in our study, higher AIS scores were more often achieved by respondents with primary education, while other studies indicate that patients with higher education achieve better results [3,38,39,40]. The literature also indicates a correlation between education and the degree of increase in health-related behaviours [13,41], which was not observed in our studies. It was observed that people on dialysis with diabetes showed a higher intensity of health behaviours, especially in terms of proper eating habits, preventive behaviours, and health practices. This may be due to a greater emphasis on health education in the case of diabetes, which is offered in Poland due to the existence of a separate medical profession—a diabetes educator [42].

The literature on the subject highlights the significant relationship between the level of disease acceptance and the intensity of patients’ health-related behaviours. This study confirmed that patients with a higher level of disease acceptance are more willing to engage in positive health behaviours regarding proper eating habits, health practices, positive mental attitudes, and preventive behaviours. Additionally, acceptance can reduce feelings of anxiety and depression, which translates into better mental and physical well-being [3,6,11,30]. Our observations are confirmed by other studies analysing patients with various chronic diseases [29,33,38,43]. The study of patients with liver cirrhosis conducted by Krzyżanowska et al. did not show any correlation between disease acceptance and health behaviours [44]. However, Kurpas et al. found only one significant association, in which higher disease acceptance was associated with a lower frequency of preventive behaviours [45]. In this context, it is worth emphasising that some authors note that higher acceptance of the disease may correlate with more passive strategies for coping with the disease [46]. Passive coping strategies, such as avoidance, withdrawal, or inaction in response to medical recommendations, may stem from the belief that the disease is beyond one’s control. Perhaps the choice of passive coping strategies by patients who accept their illness occurs in situations where effective medical interventions are unavailable. A patient with ESRD requires dialysis for life unless they receive a kidney transplant. However, this procedure is not always available due to the limited number of donors. Nevertheless, as previously presented results show, this trend may not apply to all diseases. What is possibly more important is the conclusion that the negative relationship between illness acceptance and health behaviours observed by Kurpas et al. may be reversed in some specific clinical populations [45].

Understanding the role of disease acceptance in the context of end-stage renal disease is critical to clinical practice. The research results highlight the complex network of factors influencing disease acceptance and health behaviours, suggesting that interventions aimed at increasing acceptance and modifying health behaviours should take them into account. Supporting patients in accepting their disease may lead to better motivation to adhere to treatment recommendations. It seems necessary to introduce targeted social campaigns and increase access to psychological consultations in order to increase the level of acceptance of the disease in various social groups. Educational programs that increase patients’ knowledge about their condition and indicate the benefits of following medical recommendations can significantly influence the level of acceptance [26,29,34]. Therefore, every effort should be made to ensure that patients learn and understand their disease as soon as possible and become familiar with the challenges it poses. The concept of a “Peer Support Program on Self-Management in Patients with End-Stage Renal Disease Undergoing Haemodialysis” appears in the literature, which may significantly increase disease acceptance and adherence to health behaviours by patients. The authors emphasise that this program should be implemented at an early stage of treatment, which may contribute to patients’ better adaptation to the challenges of haemodialysis [47]. Early acceptance of the disease may promote better adherence to health behaviours.

The limitations of this study include its single-centre nature and the relatively small number of participants (105 patients) who came mainly from urban areas, which may limit the generalisation of the results to the entire population of patients with kidney disease. Nevertheless, in the context of similar studies, certain consistencies were noted that may characterise this group. It is advisable to conduct further research with a larger sample to obtain more representative results.

## 5. Conclusions

The level of disease acceptance plays a critical role in shaping the health behaviours of patients with end-stage kidney disease. Higher levels of acceptance are positively associated with improved adherence to health recommendations and a more proactive approach to self-care. Disease acceptance, particularly in the context of end-stage kidney disease treated with haemodialysis, represents a complex and multifactorial construct influenced by factors such as patient age, disease duration, and the length of haemodialysis treatment. Early acceptance of the disease may promote better adherence to health-related behaviours. This underscores the necessity of implementing integrated therapeutic interventions that address psychological, sociodemographic, and clinical factors from the point of diagnosis.

## Figures and Tables

**Table 1:** Characteristics of the study group.

		N	%
Gender	female	45	42.9
	male	60	57.1
Education	primary	2	1.9
	vocational	29	27.6
	secondary	35	33.3
	higher	39	37.1
Place of residence	village	4	3.8
	town/city	101	96.2
Marital status	in a relationship	35	33.3
	single	70	66.7
Social status	unemployed	5	4.8
	employed on a contract basis	10	9.5
	employed under an employment contract	49	46.7
	pensioner	27	25.7
	annuitant	14	13.3
Frequency of adverse reactions	rare	45	42.9
	often	28	26.7
	during each treatment	8	7.6
	not applicable	24	22.9
Type of vascular access for haemodialysis	arteriovenous fistula from own vessels	67	63.8
	artificial arteriovenous fistula	6	5.7
	vascular catheter	32	30.5
Comorbidities *	diabetes	37	35.2
	arterial hypertension	49	46.7
	rheumatoid arthritis	10	9.5
	abnormal heart rate	25	23.8
	others	38	36.2
Adverse reactions during haemodialysis treatment *	headache/dizziness	13	12.4
	muscle cramps	26	24.8
	nausea/vomiting	9	8.6
	drop in blood pressure	24	22.9
	not applicable	24	22.9
	others	14	13.3
DESCRIPTIVE STATISTICS			
Age (years)	58.90 ± 12.09 (31–90)		
Duration of disease (years)	5.79 ± 3.22 (1–14)		
Duration of haemodialysis treatment (years)	3.98 ± 2.30 (1–11)		

*—multiple-choice question, N—number of respondents.

**Table 2:** Distribution of detailed results in relation to the statements included in the AIS.

Acceptance of Illness (1–5 pts.)	1		2		3		4		5		Average	SD
	N	%	N	%	N	%	N	%	N	%		
I have trouble adapting to the limitations imposed by the disease.	31	29.5	35	33.3	25	23.8	12	11.4	2	1.9	2.23	1.06
Due to my health condition, I am unable to do what I like most.	28	26.7	25	23.8	26	24.8	20	19.0	6	5.7	2.53	1.23
The disease makes me feel useless sometimes.	4	3.8	11	10.5	24	22.9	40	38.1	26	24.8	3.70	1.08
Health problems make me more dependent on others than I would like.	18	17.1	33	31.4	27	25.7	21	20.0	6	5.7	2.66	1.15
The disease makes me a burden to my family and friends.	27	25.7	34	32.4	25	23.8	12	11.4	7	6.7	2.41	1.18
My health condition makes me feel like I am not a valuable person.	4	3.8	14	13.3	21	20.0	36	34.3	30	28.6	3.70	1.13
I will never be as self-sufficient as I would like to be.	31	29.5	28	26.7	22	21.0	17	16.2	7	6.7	2.44	1.26
I think that people who are around me are often embarrassed because of my illness.	15	14.3	24	22.9	31	29.5	19	18.1	16	15.2	2.97	1.27

N—number of subjects; SD—standard deviation.

**Table 3:** Acceptance of the disease and the analysed variables.

Acceptance of Disease				
Education	Average	SD	Minimum	Maximum
Primary	32.50	0.71	32	33
Vocational	24.52	6.41	13	40
Secondary	22.37	6.39	11	35
Higher	20.97	6.23	8	31
Total	22.64	6.52	8	40
*p*	0.0203			
**Marital status**				
In a relationship	25.11	6.99	12	40
Single	21.40	5.95	8	32
Total	22.64	6.52	8	40
*p*	0.0054			
**Professional status**				
Unemployed	19.60	7.70	11	28
Employed on a contract basis	17.00	5.96	9	26
Employed under an employment contract	20.96	5.30	8	31
Pensioner	25.56	6.53	13	40
Annuitant	28.00	4.61	19	35
Total	22.64	6.52	8	40
*p*	<0.0001			

SD—standard deviation; *p*—statistical significance level.

**Table 4:** Acceptance of illness in relation to age and selected clinical variables.

		Acceptance of Disease (AIS)
Age	r	0.592
	*p*	<0.0001
Duration of the disease (years)	r	0.686
	*p*	<0.0001
Duration of the haemodialysis treatment (years)	r	0.718
	*p*	<0.0001

r—correlation coefficient; *p*—statistical significance level.

**Table 5:** Results in relation to individual categories of the HBI questionnaire.

	Increase in Health Behaviours	Proper Eating Habits	Preventive Behaviours	Positive Mental Attitude	Health Practices	HBI Stens
Average	79.11	3.32	3.33	3.36	3.18	5.16
SD	12.46	0.59	0.64	0.51	0.63	1.73
Minimum	52	2.00	2.00	2.33	1.67	2
Maximum	108	4.67	4.83	4.50	4.67	9

SD—standard deviation; HBI—Health Behaviour Inventory.

**Table 6:** Health behaviours and analysed sociodemographic variables.

The Variables			Increase in Health Behaviours	Proper Eating Habits	Preventive Behaviours	Positive Mental Attitude	Health Practices
Place of residence	village	Average	65.75	2.75	2.50	2.83	2.88
		SD	4.99	0.32	0.14	0.36	0.34
	town/city	Average	79.64	3.34	3.36	3.38	3.19
		SD	12.38	0.58	0.64	0.51	0.63
	*p*		0.0280	0.0476	0.0081	0.0366	0.3197
Marital status	in a relationship	Average	84.80	3.54	3.63	3.57	3.40
		SD	12.44	0.59	0.64	0.52	0.67
	single	Average	76.27	3.21	3.18	3.25	3.08
		SD	11.54	0.56	0.60	0.48	0.58
	*p*		0.0007	0.0052	0.0006	0.0024	0.0133
Professional status	unemployed	Average	69.80	2.97	2.87	2.90	2.90
		SD	9.23	0.41	0.48	0.47	0.62
	employed on a contract basis	Average	69.80	2.93	3.05	3.08	2.57
		SD	9.47	0.45	0.50	0.35	0.56
	employed under an employment contract	Average	76.69	3.19	3.23	3.27	3.09
		SD	11.93	0.54	0.65	0.50	0.59
	pensioner	Average	83.41	3.52	3.46	3.49	3.42
		SD	11.81	0.59	0.63	0.47	0.53
	annuitant	Average	89.29	3.77	3.77	3.74	3.60
		SD	8.75	0.49	0.56	0.47	0.58
	*p*		0.0001	0.0002	0.0075	0.0007	0.0001

SD—standard deviation; *p*—level of statistical significance.

**Table 7:** Health behaviours and comorbidities.

		Increase in Health Behaviours		Proper Eating Habits		Preventive Behaviours		Positive Mental Attitude		Health Practices	
		Average	SD	Average	SD	Average	SD	Average	SD	Average	SD
Diabetes	no	76.79	11.28	3.21	0.52	3.22	0.61	3.30	0.50	3.07	0.59
	yes	83.38	13.51	3.53	0.65	3.53	0.68	3.45	0.53	3.39	0.65
*p*		0.0090		0.0068		0.0193		0.1616		0.0110	
Arterial hypertension	no	80.95	12.72	3.37	0.59	3.43	0.66	3.41	0.55	3.28	0.65
	yes	77.02	11.94	3.27	0.58	3.21	0.61	3.29	0.46	3.07	0.58
*p*		0.1075		0.3828		0.0802		0.2144		0.0896	
Rheumatoid arthritis	no	78.56	12.27	3.30	0.58	3.30	0.62	3.34	0.50	3.16	0.63
	yes	84.40	13.68	3.53	0.66	3.58	0.82	3.53	0.62	3.42	0.53
*p*		0.1594		0.2266		0.1904		0.2495		0.2163	
Abnormal heart rate	no	80.03	12.28	3.38	0.58	3.37	0.61	3.37	0.50	3.21	0.64
	yes	76.20	12.85	3.11	0.56	3.19	0.74	3.32	0.56	3.08	0.58
*p*		0.1815		0.0442		0.2090		0.6923		0.3515	
Others	no	77.21	13.23	3.24	0.62	3.24	0.69	3.30	0.53	3.09	0.62
	yes	82.47	10.28	3.46	0.51	3.49	0.52	3.45	0.48	3.35	0.61
*p*		0.0368		0.0713		0.0578		0.1473		0.0377	

SD—standard deviation; *p*—level of statistical significance.

**Table 8:** Health behaviours, age, and selected clinical variables.

		Increase in Health Behaviours	Proper Eating Habits	Preventive Behaviours	Positive Mental Attitude	Health Practices
Age	r	0.480	0.447	0.396	0.392	0.443
	*p*	<0.0001	<0.0001	<0.0001	<0.0001	<0.0001
Duration of the disease (years)	r	0.547	0.475	0.521	0.468	0.448
	*p*	<0.0001	<0.0001	<0.0001	<0.0001	<0.0001
Duration of the haemodialysis treatment (years)	r	0.597	0.537	0.528	0.511	0.513
	*p*	<0.0001	<0.0001	<0.0001	<0.0001	<0.0001

r—correlation coefficient; *p*—statistical significance level.

**Table 9:** The relationship between the level of disease acceptance and the intensity of health behaviours.

Acceptance of Disease	Increase in Health Behaviours	Proper Eating Habits	Preventive Behaviours	Positive Mental Attitude	Health Practices
r	0.682	0.624	0.550	0.604	0.616
*p*	<0.0001	<0.0001	<0.0001	<0.0001	<0.0001

r—correlation coefficient; *p*—significance level.

## Data Availability

Dataset available upon request from the authors.
